# Correlations between different protein species of oral rinse MMP‐8 and patient‐related factors

**DOI:** 10.1002/cre2.803

**Published:** 2023-10-25

**Authors:** Jussi M. Leppilahti, Taina Tervahartiala, Hannu Kautiainen, Ismo Räisänen, Minna‐Maija Ahonen, Veli‐Jukka Uitto, Timo Sorsa, Päivi Mäntylä

**Affiliations:** ^1^ Research Unit of Population Health, Faculty of Medicine University of Oulu Oulu Finland; ^2^ Department of Oral and Maxillofacial Diseases University of Helsinki and Helsinki University Hospital Helsinki Finland; ^3^ Primary Health Care Unit Kuopio University Hospital Kuopio Finland; ^4^ Folkhälsan Research Center Helsinki Finland; ^5^ Unit of Dental Health Care Services Keski‐Uudenmaan hyvinvointialue (KEUSOTE) Hyvinkää Finland; ^6^ Division of Periodontology, Department of Dental Medicine Karolinska Institutet Stockholm Sweden; ^7^ Institute of Dentistry University of Eastern Finland Kuopio Finland; ^8^ Oral and Maxillofacial Diseases Kuopio University Hospital Kuopio Finland

**Keywords:** immunoassay, leukocyte elastase, matrix metalloproteinase 8, mouthwashes, periodontitis, point‐of‐care testing

## Abstract

**Objectives:**

The aim of this study is to examine correlations between different oral rinse matrix metalloproteinase (MMP)‐8 protein species in western blot (WB) analysis, quantitative MMP‐8 measurements, and patient‐related factors. Elevated activated MMP‐8 (aMMP‐8) associate with periodontitis and a diagnostic point‐of‐care technology has been developed based on aMMP‐8. In WB, different MMP‐8 protein species can be analyzed. Relative abundancy of fragmented 20–25 kDa forms in WB has been associated with and reflects MMP‐8 activation and related fragmentation and elevated quantitative aMMP‐8 measurements.

**Material and Methods:**

A random sample of 192 participants from a periodontal disease screening study was used for this study. Oral rinse samples for biomarker analyses were collected before clinical periodontal examinations. aMMP‐8 immunofluorometric (IFMA) and WB analysis (utilizing the same monoclonal antibody, 8708), polymorphonuclear leukocyte (PMN) elastase activity test and tissue inhibitor of metalloproteinases (TIMP)‐1 ELISA levels were performed from the oral rinse samples. Distinct MMP‐8 protein species were differentiated in the WB analysis. Principal component (PC) analysis was conducted to explore correlation patterns between the different species. Adjusted correlation analysis between the extracted PCs of WB and aMMP‐8 IFMA levels and multilevel regression analysis were conducted to explore if the other periodontal disease‐related biomarkers and clinical surrogate measures and patient‐related factors are co‐variating with the extracted components.

**Results:**

Distinct correlation patterns between the MMP‐8 protein species were observed. The first four PCs explained 89% of the whole variance in PC analysis. Statistically significant correlation (*p* < 0.05) were observed as follows: PC1 positively with 21 kDa (*r* = .69) and 25 kDa fragments (*r* = .55) and negatively with 150 kDa complexes (*r* = −.46). PC2 correlated with 45 (*r* = .70) and 55 kDa (*r* = .65) activated forms, PC3 with 70–80 kDa latent proforms (*r* = .63) and 90–100 kDa complexes (*r* = .67), and PC4 with 35 kDa fragments (*r* = .81). There were significant correlations between quantitative (IFMA) aMMP‐8 measurements and PC1 (*p* < 0.001), PC2 (<0.05) and PC3 (<0.05) but not with PC4. In multilevel regression models age, PMN elastase activity, TIMP‐1 levels, and a number of 4–5 mm periodontal pockets were associated with PC1, nonsmoking with PC2, age and PMN elastase activity with PC3, and age and smoking with PC4.

**Conclusions:**

Relative abundancy of fragmented 21–25 kDa protein species was correlated with the quantitative aMMP‐8 (IFMA) measurements, which is in line with previous results. Different patient‐related factors (smoking, age, proteolytic activity) may modify the formation of different MMP‐8 protein species in oral rinse samples and may cause variability in quantitative aMMP‐8 measurement.

## 1 INTRODUCTION

Matrix metalloproteinase (MMP)‐8 (collagenase‐2, neutrophil collagenase) is recognized as among the most interesting biomarkers in periodontal diagnostics. Collagenase activity in diseased periodontal tissues is mainly contributed by MMP‐8 instead of other collagenases MMP‐1 and ‐13, and especially active MMP‐8 but not total MMP‐8 is associated with and reflects periods of active connective tissue destruction and a clinical diagnosis of periodontitis (Romanelli et al., 1999). MMP‐8 is not only involved in pathologic tissue destruction but also in physiologic tissue modeling and remodeling as well as tissue repair (Nwomeh et al., 1999; Pirilä et al., 2007). However, elevated oral fluid MMP‐8 levels have been associated with periodontal disease and MMP‐8 levels have been shown to correlate with periodontal clinical surrogate measures (Arias‐Bujanda et al., 2019, 2020; Sorsa et al., 2016), and the research has proceeded from the earlier studies in the late 1970s (Uitto et al., 1978) to development (Heikkinen et al., 2016; Mäntylä et al., 2003, 2006; Sorsa et al., 1999) and to the validations of point‐of‐care (aMMP‐8 POCT)‐ diagnostic tests (Deng et al., 2021, 2022; Lähteenmäki et al., 2022; Lorenz et al., 2017; Räisänen et al., 2021; Sorsa et al., 2020).

Different environmental factors and conditions may modify oral fluid MMP‐8 levels, for example, smoking, diabetes, as well as other systemic inflammatory diseases and conditions (Deng et al., 2021; Grigoriadis et al., 2019; Lahdentausta et al., 2019; Räisänen et al., 2021; Rautava et al., 2020; Sorsa et al., 2016). There have been also significant variations between different quantitative immunological MMP‐8 assays that are probably caused by variations in antibody sensitivities and specificities on different protein forms of MMP‐8 (Gursoy et al., 2010; Leppilahti et al., 2011; Sorsa et al., 2010, 2021, 2022).

MMP regulation is a complex process involving synthesis, secretion, activation, and inhibition (Romanelli et al., 1999). When neutrophils are recruited to a site of inflammation, they release latent MMP‐8 stored in specific granules. Removal of the prodomain by enzymes of host or microbial origin results in a reduction of the molecular mass of the latent proMMPs (DeCarlo et al., 1997; Ding et al., 1995; Romanelli et al., 1999; Sorsa et al., 1992, 1995). Different proteolytic enzymes cleave at different sites in the proenzyme domain, generating different sizes of active and fragmented enzymes with different levels of enzyme activity (Romanelli et al., 1999). There was some discrepancy in the western blot (WB) molecular weights of latent and active MMP‐8 described in the early literature with values ranging from 85 kDa (Knauper et al., 1990) to 58 kDa (Uitto et al., 1990; van Wart, 1992) reported from polymorphonuclear leukocytes (PMN) preparations or from gingival crevicular fluid (GCF) samples. Different sample materials, preparations, and procedures may, at least partly, explain the differences in molecular sizes and forms, and the antibodies used in immunoblot may also give variant results.

Bacterial proteinases present in supra‐ and subgingival plaque can activate and process the PMN‐type MMP‐8 to less glycosylated 40–60 kDa forms. Species ≤30 kDa are regarded as fragments (Kiili et al., 2002). Romanelli et al. (1999) found these processed forms of MMP‐8 from GCF, which was sampled by oral rinse method from periodontitis patients. They considered the smaller MMP‐8 forms (around 60 kDa) as biologically important and hypothesized, that these species may be efficiently activated or even superactivated fragmented species. Especially *Porphyromonas gingivalis* and *Treponema denticola* can catalyze the superactivation and fragmentation of MMP‐8 by gingipains and dentilisin (Ding et al., 1995, 1996, 1997; Nieminen et al., 2018; Romanelli et al., 1999; Sorsa et al., 1992, 1995). In contrast, high molecular weight immunoreactivity (>100 kDa) is regarded as MMP‐8 complexed to its endogenous inhibitors α‐2‐macroglobulin and tissue inhibitors of metalloproteinases (Kiili et al., 2002).

The aim of this study is to further explore correlations between different MMP‐8 protein species in WB analysis and quantitative IFMA measurements, patient‐related factors, and periodontal disease surrogates.

## 2 MATERIAL AND METHODS

### 2.1 Patients

The clinical data of this study was originally designed and collected for a study testing the utility of neutrophil elastase activity test for screening periodontitis patients. Randomly selected 214 patients, who sought general dentists' treatment in public oral health services in the cities of Helsinki and Vantaa in Finland, were included in this cross‐sectional study. Patients in need of antibiotic prophylaxis or suffering a contagious diseases were excluded. The study protocol has been presented in detail previously by Leppilahti et al. (2011). Concisely, oral rinse sampling for the PMN elastase activity test and oral examination, comprised of measurements of pocket probing depths (PPD) and of bleeding on probing (BOP) by a Florida probe carried out by two calibrated general dentists, were performed for all included patients. Clinical periodontal diagnoses were assessed according to the 1999 classification. Background characteristics were recorded by questionnaires before clinical examinations. All patients gave an informed consent. Ethical committees of the Institute of Dentistry, University of Helsinki, and Helsinki University Central Hospital accepted the study protocol.

### 2.2 Oral rinse sampling and elastase activity test

Oral rinse samples for PMN elastase activity testing were collected before clinical examination. By means of a disposable plastic pipette, 1 mL of tap water was placed into the patient's mouth, and after rinsing for 1 min the rinse was collected into a tube (Nieminen et al., 1993; Uitto et al., 1996). Testing for PMN elastase activity was performed immediately after the oral rinse sample collection. The rest of the sample was immediately frozen for further analyses (Uitto et al., 1996).

### 2.3 MMP‐8 analyses

Quantitative aMMP‐8 (IFMA) and TIMP‐1 (ELISA) measurements were performed from the same oral rinse sample as the PMN elastase activity test. The results of aMMP‐8 IFMA and TIMP‐1 measurements were reported previously together with PMN elastase activity test results (Leppilahti et al., 2011). aMMP‐8 IFMA measurements followed the same protocol described by Hanemaaijer et al. (1997) (Hanemaaijer et al., 1997). Briefly, the monoclonal aMMP‐8‐specific antibodies 8708 and 8706 were used as a catching antibody and a tracer antibody, respectively. The tracer antibody was labeled using europium‐chelate (Hemmilä et al., 1984). Samples were diluted in assay buffer and incubated for 1 h, followed by incubation for 1 h with tracer antibody. An enhancement solution was added, and after 5 min fluorescence was measured using a 1234 Delfia Research Fluorometer (Wallac).

From the 214 patients originally included, 192 frozen oral rinse samples were available for further WB for this study with the same principles described previously (Hanemaaijer et al., 1997; Kiili et al., 2002; Lauhio et al., 1994).

The molecular forms of MMP‐8 were detected by using a modified enhanced chemiluminescence (ECL) WB kit according to the protocol recommended by the manufacturer (GE Healthcare) as described earlier (Gürsoy et al., 2018). Briefly: the oral rinse samples were mixed with Laemmli's buffer without any reducing reagents and heated for 5 min, followed by protein separation with 11% sodium dodecyl sulfate (SDS)–polyacrylamide gels. After electrophoresis, the proteins were electrotransferred onto nitrocellulose membranes Protran (Whatman GmbH, Dassel, Germany). Nonspecific binding was blocked with 5% milk powder (Valio Ltd.) in TBS‐T buffer (10 mM Tris‐HCl, pH 7.5, containing 22 mM NaCl and 0.05% Triton‐X) for 1 h. The membranes were incubated with monoclonal primary antibodies anti‐aMMP‐8 as by IFMA methods overnight, and then with horseradish peroxidase‐linked secondary antibody (GE Healthcare) for 1 h. The membranes were washed four times in TBS‐T buffer between each step for 15 min. The proteins were visualized using the ECL system and scanned and analyzed using the GS‐700 Imaging Densitometer Scanner (Bio‐Rad) and Bio‐Rad Quantity One program.

The analyses utilized the same catching antibody (8708) of the IFMA method for the identification of different molecular forms and species of MMP‐8 by scanning image analysis. We regarded each MMP‐8 molecular form finding as positive if a band of different molecular weights was observable and densitometric units showed value > 0. Because we wanted to study the correlation between quantitative MMP‐8 levels and different protein forms and species in WB of the same oral rinse sample, it was more reasonable to use the same monoclonal antibody (catching ab 8708) utilized in the quantitative measurement and in the WB.

### 2.4 Statistical methods

WB analysis is a semiquantitative immunological analysis method, and direct quantitative comparison between samples that are not analyzed in the same batch of immunoblotting and electrophoresis, is not advisable. However, the quantitative measurement and comparison of protein weights between samples within the same batch is possible. The patient (sample) specific relative percentual quantities of different protein weights in WB were calculated and further Van der Waerden rank‐based normalization was performed to calculate correlations between all samples. Principal component (PC) analysis (Soloman & Sawilowsky, 2009) was conducted to explore correlation patterns of different WB MMP‐8 protein forms. The polychoric correlation method and rotated Varimax method were used to extract PCs.

Sidak's correlations between PCs and quantitative MMP‐8 IFMA levels were calculated by adjusting for age, gender, and smoking.

Multilevel regression analysis was conducted to test associations between WB principal components and periodontal disease‐related surrogate measures and patient‐related factors.

Stata 16.0 (StataCorp LP) was used for all statistical analyses.

## 3 RESULTS

### 3.1 Patient characteristics

Background characteristics of study participants in relation to oral rinse aMMP‐8 IFMA levels are described in Table 1. aMMP‐8 levels correlated significantly with increasing elastase activity, BOP measures, and TIMP‐1 levels.

**Table 1:** Patient characteristics in relation to activated matrix metalloproteinase‐8 (aMMP‐8) immunofluorometric (IFMA) levels (tertiles).

	Tertiles of MMP‐8 IFMA levels			
	I	II	III	*p*
	*n* = 64	*n* = 64	*n* = 64	
Women (yes), *n* (%)	46 (73)	42 (67)	39 (61)	0.15
Age (year), mean (SD)	56 (8)	57 (7)	58 (8)	0.20
Diabetes, (yes), *n* (%)	2 (3)	7 (11)	6 (9)	0.25
Smoking, (yes), *n* (%)	18 (29)	15 (24)	16(25)	0.65
Medication prescription (yes)	35 (56)	36 (57)	43 (67)	0.18
Clinical diagnosis, *n* (%)				0.58
Periodontally healthy	10 (16)	13 (21)	8 (13)	
Gingivitis	26 (41)	27 (43)	24 (38)	
Periodontitis	27 (43)	23 (37)	32 (50)	
Number of teeth, mean (SD)	24 (5.2)	25 (4.1)	24 (5.0)	0.61
BOP%, mean (SD)	**9.6** (**7.3)**	**13.1** (**12.9)**	**13.5** (**9.3)**	**0.009**
Number of sites PPD 4–5 mm, mean (SD)	4.9 (7.3)	4.5 (9.8)	5.8 (8.4)	0.50
Number of sites PPD ≥ 6 mm, mean (SD)	1.1 (3.0)	1.1 (3.6)	2.2 (4.9)	0.13
MMP‐8 IFMA (ng/mL), median (IQR)	**102 (52.2)**	**481 (339)**	**1170 (988)**	**<0.001**
TIMP‐1 ELISA (ng/mL), median (IQR)	**174** (**118; 252)**	**168** (**114; 230)**	**111** (**60; 205)**	**0.002**
Elastase activity (yes), *n* (%)	**3** (**5)**	**23** (**37)**	**45** (**70)**	**<0.001**

*Note*: Data are expressed as number of study participants and respective percentages or mean and SD in relation to demographics and clinical parameters and median/IQR of skewed data of TIMP‐1. Statistically significant (<.05 level) differences between the tertile groups are bolded.

Abbreviations: BOP, bleeding on probing; IQR, interquartile range; PPD, pocket probing depth; SD, standard deviation.

#### 3.1.1 Western immunoblot analysis of proMMP‐8 activation in oral rinse samples

Different protein forms >150 kDa complexes, complexes (or preproforms) in the range from 90 to 100 kDa, 70–80 kDa latent (PMN) proforms, two activated forms/bands in 45 and 55 kDa, and 35, 25, 21 kDa fragmented forms and their relative (%) semiquantitative measures were observed and defined in WB.

**Figure 1:**
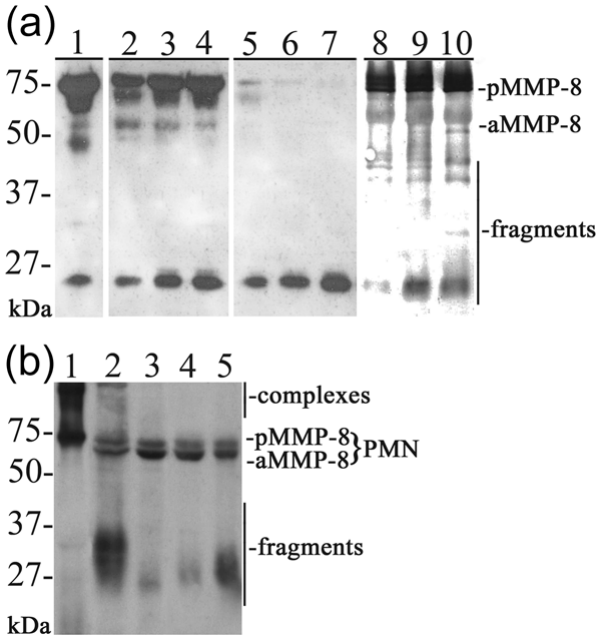
Western blot analysis with (a) recombinant human proMMP‐8 activated by NaOCl, 4‐Aminophenylmercuric acetate (APMA) and *Treponema denticola* dentilisin, and (b) representative oral rinse samples. (a) Lane 1: ProMMP‐8 100 ng; lanes 2–4: as lane 1 but activated by 100 µm NaOCl; lanes 5–7: as lane 1 but activated by 0.5 mM APMA; lanes 8–10 as lane 1 but activated by *Porphyromonas gingivalis* and *T. denticola* dentilisin. (b) Lane 1: sample from periodontally healthy with latent recombinant proform (pMMP‐8); lanes 2–5: oral rinse samples of periodontitis patients in which activated (aMMP‐8), fragmented and complex formation can be observed in addition to the proform (pMMP‐8). pMMP‐8 indicates 70–80 kDa proMMP‐8, aMMP‐8 indicates activated 40–60 kDa MMP‐8 species, and fragments indicate 21–35 kDa fragmented low molecular size MMP‐8 species. Different weight complex formation can be observed (>90 kDa). The molecular weight scale of the western blot is indicated on the left.

Figure 1 shows the immunoblot analysis of recombinant latent proMMP‐8 activated by NaOCl (Saari et al., 1990), organomercurial 4‐Aminophenylmercuric acetate (APMA) (Saari et al., 1990), treponemal chymotrypsin‐like protease (dentilisin) (Nieminen et al., 2018) (Figure 1a), and representative analysis of oral rinse samples from healthy participants and periodontitis patients (Figure 1b). All these known proMMP‐8 activators activated proMMP‐8 to lower molecular weight forms (40–60 kDa) and related low molecular size (25–40 kDa) fragments. However, if latent MMP‐8 was activated with a protease (dentilisin), a more versatile set of different‐sized species can be observed in the range from 40 to 60 kDa (indicated as aMMP‐8) and from 25 to 35 kDa (fragments) (Figure 1a, lanes 8–10) corresponding with the biological/in vivo oral rinse samples (Figure 1b, lanes 2–5) of periodontitis patients. Latent 70–80 kDa proforms of MMP‐8 could be detected in the samples of periodontally healthy mouths (Figure 1b, lane 1), lower molecular size fragmented (25–35 kDa), and activated forms (40–60 kDa) in the samples from periodontitis patients (Figure 1b, lanes 2–5).

### 3.2 Correlation patterns in principal component analysis

**Figure 2:**
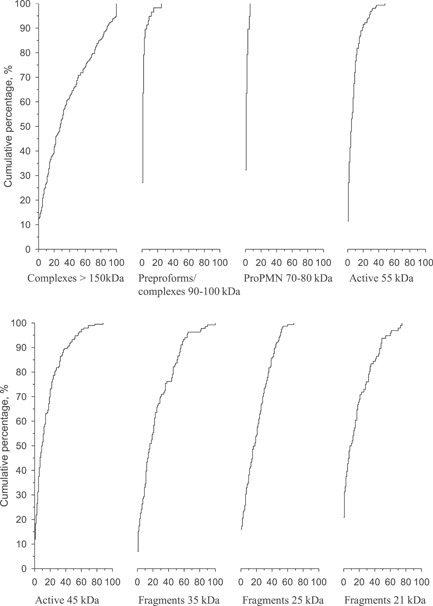
Cumulative frequency distributions of different matrix metalloproteinase‐8 protein species in western blot analyses.

Cumulative frequency distributions (CFD) of different MMP‐8 species (percentual relative quantity) in WB express that the data were right‐skewed (Figure 2). In the CFD, the frequencies of (relative quantities of) each MMP‐8 species are expressed cumulatively. The steeper the curve is in CFD, the more infrequent/rare the studied MMP‐8 species are in comparison to other MMP‐8 species. Fragmented and 150 kDa complex forms were the most abundant MMP‐8 species while the levels of 90–100 kDa complexes, 70–80 kDa proforms, and 45 and 55 kDa activated forms could not be detected in many samples. For this reason, the relative quantities of different protein weights were transformed with van der Waerden rank‐based normalization to conduct principal component analysis.

**Figure 3:**
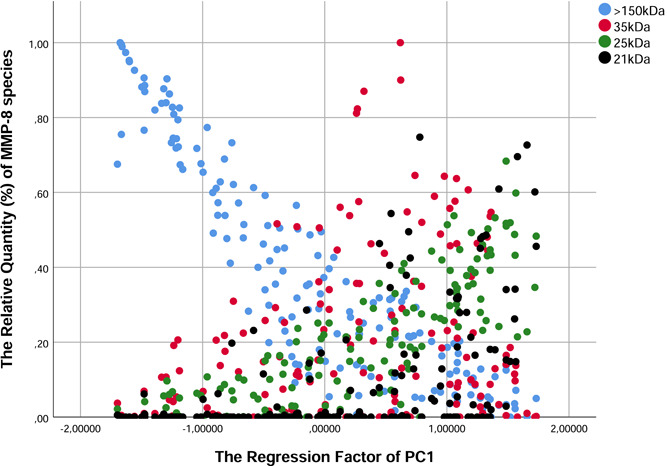
Scatterplot illustrating the correlations between the relative quantity (%) of different matrix metalloproteinase‐8 (MMP‐8) species and the regression factor of the first component (PC1) in principal component (PC) analysis.

In the PC analysis, distinct correlation patterns between MMP‐8 molecular weights were observed (Table 2). The four first components explained 89% of the whole variance and there was no significant overlap between the components. PC1 correlated with fragments (21, 25 kDa), PC2 with the activated forms (45 and 55 kDa), and PC3 with proforms (70–80 kDa) and complexes (90–100 kDa). PC4 correlated with the fragment 35 kDa. There were significant negative correlations between 21 and 25 kDa fragments and 150 complexes, and the negative correlation of 150 complexes was loaded on the PC1 (Figure 3).

**Table 2:** Correlation coefficients between the extracted principal components (PCs 1–4) and different matrix metalloproteinase‐8 (MMP‐8) protein species in western blot.^a^

MMP‐8 molecular forms	PC1	PC2	PC3	PC4	Unexplained
Fragments 21 kDa	**0.69** a	−0.09	0.08	−0.36	0.06
Fragments 25 kDa	**0.55** a	−0.05	0.08	0.24	0.17
Fragments 35 kDa	−0.07	−0.07	0.07	**0.81** a	0.04
Active 45 kDa	−0.06	**0.70** a	−0.09	−0.04	0.08
Active 55 kDa	0.01	**0.65** a	0.17	−0.04	0.09
pro PMN 70–80 kDa	0.05	0.08	**0.63** a	0,10	0.19
Preproforms/complexes 90–100 kDa	0.03	−0.01	**0.67** a	0.02	0.18
Complexes > 150 kDa	**−0.46** a	−0.25	0.32	−0.36	0.07

Abbreviation: PMN, polymorphonuclear leukocyte.

a Principal components (PC) 1–4 explain 89% of the whole variance, Quartile ranks used for expressing molecular form quantity. Statistically significant (<0.05 level) correlations (polychoric) are bolded.

### 3.3 Correlation between PCs and MMP‐8

Correlation coefficients adjusted by age, gender, and smoking between quantitative IFMA aMMP‐8 measurements and different PCs were calculated (Table 3). There was significant (*p* < 0.001) correlation between PC1 (fragments 21 and 25 kDa) and IFMA aMMP‐8. Also, PC3 correlated significantly (*p* < 0.05) with IFMA aMMP‐8. Interestingly, there was a negative significant correlation (*p* < 0.05) between IFMA and 45 and 55 kDa protein weights (activated forms).

**Table 3:** Correlation between the principal components (PC) and matrix metalloproteinase‐8 (MMP‐8) immunofluorometric measurements.

	MMP‐8 (95% CI)
PC1	0.34 (0.19 to 0.50)***
PC2	−0.17 (−0.29 to −0.06)**
PC3	0.17 (0.04 to 0.30)**
PC4	0.03 (−0.08 to 0.14)

*Note*: Sidak's Correlation coefficients are adjusted by age, gender, and smoking.

Abbreviation: CI, confidence interval.

*** *p* < 0.001

** *p* < 0.05.

### 3.4 Multilevel modeling of PCs

In multilevel models (Table 3), age and the number of sites with PPD 4–5 mm were negatively correlated with PC1, while elastase activity and TIMP‐1 levels were correlating positively with PC1. Smoking was associated negatively with PC2, whereas PC4 was associated positively with smoking. Age and the number of teeth were correlating negatively with PC4. Age correlated positively with PC3.

## 4 DISCUSSION

We found clear correlation patterns between the different MMP‐8 WB molecular forms in PC analysis. The first four PCs explained 89% of the whole variance. For example, the fragmented 21 and 25 kDa forms were correlating negatively with the >150 kDa complexes. This suggests that MMP‐8 appears more in fragmented forms in certain samples and in other cases MMP‐8 is compounded into large molecular size complexes. Fragmented forms correlated strongly with the quantitative IFMA aMMP‐8 levels, which has also been observed earlier in other data sets utilizing independent polyclonal anti‐MMP‐8 antibodies (Buduneli et al., 2011; Gürsoy et al., 2018). In multilevel models, different PCs of MMP‐8 protein species were associated with smoking, age, PMN elastase activity, the number of teeth, and the number of moderately deep periodontal pockets (4–5 mm). Especially fragmented low molecular size MMP‐8 species and PMN elastase activity are surrogates and biomarkers of periodontal disease activity and collagenolytic and proteolytic inflammatory burden (Buduneli et al., 2011; Kiili et al., 2002; Leppilahti et al., 2011; Romanelli et al., 1999; Uitto et al., 1990). To the best of our knowledge, this is the first study analyzing correlations between WB and quantitative MMP‐8 measurement utilizing the same antibody that is also used in the aMMP‐8 point‐of‐care test (Deng et al., 2021, 2022; Lähteenmäki et al., 2022; Sorsa et al., 2020), and further on correlation between the different protein species in WB and patient‐related factors and clinical surrogates.

The different MMP‐8 molecular weights in WB have been reported previously (Hanemaaijer et al., 1997; Kiili et al., 2002; Knauper et al., 1990; Knäuper et al., 1993; Owen et al., 2004; Sorsa et al., 1985, 1988). Hanemaaijer et al. (1997) showed that purified PMN MMP‐8 had a molecular weight of 75–85 kDa, while endothelial and fibroblastic cells and fibroblast expressed de novo (“mesenchymal”) MMP‐8 with molecular weight around 50 kDa. The difference in the molecular weights of PMN and mesenchymal MMP‐8 was explained with glycosylation of PMN‐derived MMP‐8. After deglycosylation, PMN MMP‐8 expressed four different bands in WB (70, 65, 50, and 45 kDa) and the 50 kDa molecular form corresponded the weight of nonglycosylated mesenchymal derived MMP‐8. Knäuper et al. (1990, 1993) reported similar results comprising molecular weights of 84 and 64 kDa for purified PMN proMMP‐8 and activated form, and 53 kDa for deglycosylated proPMN MMP‐8.

Autoproteolytic fragmentation of MMP‐8 has been described previously (Knäuper et al., 1993). Activated MMP‐8 (64 kDa) was autofragmented into a 40 and 27 kDa molecular form after 24 h incubation at 37° temperature. The fragmented MMP‐8 was not able to cleave collagen I but the 40 kDa form had a similar proteolytic activity profile with the intact activated 64 kDa MMP‐8 (Knäuper et al., 1993). The 27 kDa fragment had lost proteolytic activity after fragmentation. The 35 kDa band observed in our study was close to 40 kDa and could represent the autofragmented form of MMP‐8. The precise mechanism producing lighter 21 and 25 kDa forms is not well known. However, the smaller sizes of MMP‐8 species found in dental plaque are regarded to be due to proteolytic fragmentation by bacterial proteases (Kiili et al., 2002; Romanelli et al., 1999; Sorsa et al., 1995). *T. denticola* and other pathogens involved in dysbiosis with proteases such as dentilisin and gingipains can activate pMMP‐8 (Nieminen et al., 2018; Sorsa et al. 1992, 1995). Romanelli et al. (1999) found these processed forms (55–60 kDa) of MMP‐8 from GCF, which was sampled by oral rinse method from periodontitis patients. They considered these smaller MMP‐8 forms as biologically important and hypothesized, that these species may be efficiently activated or even superactivated species. Especially *P. gingivalis* and *T. denticola* could catalyze the superactivation of MMP‐8 by their gingipains and dentilisins (Nieminen et al., 2018; Romanelli et al., 1999; Sorsa et al. 1992, 1995). Making this even more complex, Owen et al. (2004) have observed in the WB analysis of activated human PMN plasma membranes three major (85, 65, and 30 kDa) and three minor (110, 80, and 45 kDa) molecular forms of MMP‐8. They speculated that the lighter 30 and 45 kDa forms are proteolytically cleaved inactive forms of MMP‐8.

In the present study, eight different bands were defined indicating the molecular weights of >150, 90–100, 70–80, 55, 45, 35, 25, and 21 kDa corresponding, according to the previous literature, complex forms, different PMN proforms, activated forms, and three different fragmented forms. In this study, there was a distinct broader band around 90–100 kDa indicating probably MMP‐8 complexes or so‐called preproforms as suggested by Määttä et al. (2006). It is possible that the 90–100 kDa band includes also MMP‐8 bound with TIMP‐1 (28 kDa) as discussed below (Määttä et al., 2006; Owen et al., 2004). Such multiple molecular forms have also been identified in urine from diabetic nephropathic patients (Lauhio et al., 2008). However, only a minority of patients expressed a detectable band around 90–100 kDa (Figure 2).

The exact content of heavy complexes including MMP‐8 and the mechanism leading to complex formation in oral fluid samples is not well known either. Reasonable explanations for heavy complexes could be that TIMP‐1 is combined with MMP‐8 (Määttä et al., 2006). The molecular weight of TIMP‐1 is 28 kDa and if added to the 64 kDa weight of activated MMP‐8 it sums up to around 90 kDa. Owen et al. (2004) also showed that TIMP‐1 can be bound to the cellular membrane of PMN with unknown receptors and membrane‐bound TIMP‐1 that can further bind to MMP‐8 via their COOH‐terminal hemopexin domains (Owen et al., 2004; Wang et al., 2019). The membrane‐bound TIMP‐1–MMP‐8 complex had interesting counterintuitive functional properties to promote pericellular proteolysis. TIMP‐1 was also shown to enable MMP‐8 and MMP‐9, anchoring into neutrophil extracellular traps (NETs), extracellular DNA fibers restricting bacterial invasion and with bactericidal effects, facilitating proteolysis (Wang et al., 2019). These membrane‐bound or NET‐associated MMP‐8‐ TIMP1 complexes could explain the heavy MMP‐8 complexes in WB possibly also including other unknown components. TIMP‐1 (28 kDa) and proMMP‐8 (85 kDa) molecular weights sum up to 113 kDa, which is near 110 kDa.

Activated protein species could be observed in WB around 45 and 55 kDa. In some cases, clearly, distinct bands cannot be observed, but diffuse signals are observed in the range from 40 to 60 kDa corresponding to the experimentally activated latent MMP‐8 with dentilisin (Figure 1a, lanes 8–10). In some studies, mesenchymal MMP‐8 (experimental samples from endothelial cells and fibroblasts) is observed around 40–50 kDa as described above (Hanemaaijer et al., 1997). However, it can be assumed that MMP‐8 in oral rinse samples is mainly derived from PMN leukocytes, as the cell type represents around 90% of cells in the GCF (Delima & van Dyke, 2003). So, it is more reasonable to assume that the 45 kDa aMMP‐8 species are proteolytically cleaved PMN‐derived MMP‐8. However, in the limits of the methods used in this study, we cannot verify, whether MMP‐8 is of mesenchymal or PMN origin.

The PMN elastase activity was associated with the relative quantity of fragmented forms of MMP‐8 and with aMMP‐8 levels measured by IFMA and reflected periodontal inflammatory burden. PMN elastase is a neutrophil‐derived enzyme and PMN elastase activity probably indicates enzymatic activity eventually promoting and enhancing MMP‐8 fragmentation. PMN elastase does not directly cleave MMP‐8 but is strongly correlating with cathepsin G and myeloperoxidase, which are directly related to proteolytic or oxidative activation and fragmentation of MMP‐ 8 and may also explain the correlation with fragmentation (Buduneli et al., 2011; Hernández et al., 2010; Leppilahti et al., 2014; Owen et al., 2004; Wang et al., 2019).

An interesting new finding was the association between age and different molecular forms of MMP‐8 in WB. The PC1, which was correlating positively with the fragmented forms but negatively with the complexes had a negative significant correlation with age in the multilevel model. It seems that in younger participants, the relative quantity of fragmented MMP‐8 is higher, while in older subjects MMP‐8 is compounded into the heavy complexes. In turn, PMN leukocyte molecular forms (PC3) correlated positively with participants' age (Table 4). There are several possible explanations for the observed associations. Age is a well‐known confounding factor of periodontitis prevalence, extent, and severity. We have not performed additional post hoc analysis that would consider age and periodontitis as confounding factors, but it can be speculated if periodontitis prevalence and severity would have modifying effects on MMP‐8 turnover and processing in oral fluids. Deeper periodontal pockets correlate with the dysbiosis‐associated periodontal key pathogens, which express MMP‐8 activating enzymes gingipains (*P. gingivalis*), trypsins, and dentilisin (*T. denticola*) as described above (DeCarlo et al., 1997; Nieminen et al., 2018; Sorsa et al., 1992, 1995).

**Table 4:** Multilevel models of the western blot principal components (PC).

	Coefficient	SE	*t*	*p* > (*t*)	95% CI	
PC1						
Age	**−0.35**	**0.07**	**−4.95**	**0.000**	**−0.49**	**−0.21**
Gender	0.06	0.06	0.91	0.36	−0.07	0.19
Smoking	−0.11	0.07	−1.53	0.13	−0.24	0.03
Number of teeth	−0.08	0.07	−1.05	0.30	−0.22	0.07
BOP%	−0.001	0.07	−0.02	0.99	−0.15	0.14
*N* of sites PPD 4–5 mm	**−0.24**	**0.11**	**−2.30**	**0.02**	**−0.45**	**−0.03**
*N* of sites PPD ≥ 6 mm	0.19	0.10	1.87	0.06	−0.01	0.39
Elastase activity	**0.42**	**0.07**	**6.29**	**0.000**	**0.29**	**0.55**
TIMP‐1 (ng/mL)	**0.20**	**0.07**	**2.91**	**0.004**	**0.06**	**0.34**
Constant	0.0002	0.06	0.00	1.0	−0.12	0.12
PC2						
Age	0.11	0.08	1.34	0.18	−0.05	0.27
Gender	−0.03	0.07	−0.37	0.71	−0.17	0.12
Smoking	**−0.21**	**0.08**	**−2.72**	**0.007**	**−0.37**	**−0.06**
Number of teeth	0.06	0.08	0.78	0.43	−0.10	0.23
BOP%	−0.06	0.08	−0.68	0.50	−0.22	0.11
*N* of sites PPD 4–5 mm	0.003	0.12	0.02	0.98	−0.24	0.24
*N* of sites PPD ≥ 6 mm	0.05	0.12	0.39	0.70	−0.18	0.28
Elastase activity	0.02	0.08	0.29	0.77	−0.13	0.17
TIMP1 ELISA	0.10	0.08	1.29	0.20	−0.05	0.25
Constant	0.01	0.07	0.14	0.89	−0.13	0.15
PC3						
Age	**0.20**	**0.08**	**2.49**	**0.01**	**0.04**	**0.37**
Gender	−0.01	0.07	−0.18	0.86	−0.16	0.13
Smoking	0.001	0.08	0.01	0.99	−0.16	0.16
*N* of teeth	0.08	0.08	0.97	0.33	−0.08	0.24
BOP%	0.02	0.08	0.21	0.84	−0.15	0.18
*N* of PPD 4–5 mm	−0.05	0.12	−0.45	0.66	−0.29	0.19
*N* of PPD ≥ 6 mm	0.08	0.12	0.70	0.48	−0.15	0.31
Elastase activity	0.14	0.08	1.88	0.06	−0.01	0.29
TIMP1 ELISA	0.08	0.08	0.99	0.33	−0.08	0.23
Constant	0.01	0.07	0.17	0.87	−0.13	0.16
PC4						
Age	**−0.18**	**0.08**	**−2.26**	**0.03**	**−0.33**	**−0.02**
Gender	0.06	0.07	0.91	0.36	−0.08	0.21
Smoking	**0.18**	**0.08**	**2.41**	**0.02**	**.03**	**0.34**
*N* of teeth	**−0.16**	**0.08**	**−2.00**	**0.05**	**−0.32**	**−0.002**
BOP%	−0.13	0.08	−1.61	0.11	−0.29	0.03
*N* of sites PPD 4–5 mm	−0.20	0.12	−1.75	0.08	−0.43	0.03
*N* of sites PPD ≥ 6 mm	0.17	0.11	1.52	0.13	−0.05	0.39
Elastase activity	0.11	0.07	1.55	0.12	−0.32	−0.26
TIMP‐1 (ng/mL)	0.09	0.08	1.19	0.24	−0.06	0.24
Constant	−0.02	0.07	−0.24	0.81	−0.15	0.12

*Note*: PC values are *z*‐normalized. Statistically significant (<0.05 level) associations are bolded.

Abbreviations: BOP, bleeding on probing; TIMP, tissue inhibitor of matrix metalloproteinases.

A negative correlation between age and TIMP‐1 levels was reported with this same data set when the association of periodontal parameters and quantitative aMMP‐8 levels was reported previously (Leppilahti et al., 2011). Significant age‐dependent association was observed in the ratio of aMMP‐8 (IFMA) and TIMP‐1 (ELISA) measurements but not in aMMP‐8 (IFMA) measures itself. Several recent clinical trials, studying the accuracy of the aMMP‐8 test, have reported that age is a significant cofactor affecting the aMMP‐8 test accuracy (Deng et al., 2021; Räisänen et al., 2021).

Migration of neutrophils toward dental biofilms and release of NETs on supragingival biofilm has been observed and it can be assumed that NETs exist also in oral rinse samples (Hirschfeld et al., 2015; Preshaw et al., 2017). On the other hand, a significant age‐related decrease in reactive oxygen species (ROS) and NET formation was observed after induction with lipopolysaccharide and interleukin‐ 8 (Hazeldine et al., 2014). It can be speculated if age‐related changes in neutrophil functions, ROS, and NET release can have effects on MMP‐8 activation and fragmentation versus complex formation also taking into account NET‐related TIMP‐1–MMP‐8 complex discussed above (Wang et al., 2019).

A strength of this study was that both quantitative aMMP‐8 (IFMA) measurements and WB analysis were performed by utilizing the same monoclonal antibody. However, it should be kept in mind that the immunofluorometric measurements as IFMA and sandwich ELISA methods utilize two monoclonal antibodies, called primary/catching and secondary/detection antibodies. In WB, only one antibody is used, and the same primary/catching antibody (8708) was utilized in this WB analysis as was used in the quantitative IFMA. This monoclonal antibody correlates well with the independent polyclonal antibody (Buduneli et al., 2011; Hernández et al., 2010), and both have been utilized to assess different forms of periodontal disease (Chen et al., 2000; Gürsoy et al., 2018; Kiili et al., 2002; Sorsa et al., 1999). The primary antibody was used because it is highly specific to catch the target, while the secondary antibody was not designed to be used for detection solely. It is also worth to keep in mind that although the antibodies are same as utilized in the commercially available aMMP‐8 point‐of‐care test (Deng et al., 2021, 2022; Lähteenmäki et al., 2022; Sorsa et al., 2020), the oral rinsing sampling method was different (Leppilahti et al., 2011), and quantitative IFMA aMMP‐8 levels are not directly comparable with quantitative aMMP‐8 test results.

In WB analyses, different molecular forms are separated with electrophoresis based on their molecular weights, and after electrophoresis, the target protein is immunologically detected and blotted with a specific antibody. Quantification of the studied molecule is based on measuring the colorimetric/fluorescence reaction of the labeled antibody. A challenge in quantitative statistical analysis of WB measurements is that there might be significant variation between WB batches, that is, samples analyzed within the same batch of electrophoresis and immunoblotting can be quantitatively compared with each other but between the batches, comparisons cannot be done. Thus, WB is called a semi‐quantitative measuring method in comparison to quantitative immunological methods such as ELISA, IFMA, and so forth. In this study, we solved this methodological problem by calculating the sample‐specific relative (%) quantities of different MMP‐8 species and we conducted further statistical analyses with these relative quantities of different molecular weights. So, it is important to keep in mind that we are speaking about the percentual share of different MMP‐8 species in oral rinse samples and their correlations with periodontitis‐related factors, not about absolute quantities of these species.

Among the weaknesses of this study is that periodontal diagnosis does not comply with the new classification; clinical attachment level was not recorded, and the disease stage and grade could not be assessed. Florida probe device was used for PPD and BOP measurements. The mean BOP percentages were surprisingly low in the study population if the same patients had deep periodontal pockets, had no periodontal treatment recently, and were seeking oral examination and treatment. Thus, it can be speculated if there eventually was a systematic error in BOP detection, even though BOP percentages were low, there was a positive correlation between BOP percentage and MMP‐8 (IFMA) levels which is in line with previous studies (Leppilahti et al., 2011; Räisänen et al., 2019). In the previous study with this same data set (Leppilahti et al., 2011), we could differentiate patients with a strong periodontal inflammatory burden, defined by an inflammatory burden index combining the information of the number of deep periodontal pockets and BOP percent, from periodontally healthy participants. However, as reported in the current study, there was solely a tendency but not significant association between IFMA aMMP‐8 tertiles and number of periodontal pockets with PPD 4–5 mm and ≥6 mm (Table 1), which differs from most previous studies focusing on correlation/association between aMMP‐8 and periodontal clinical surrogates (Rautava et al., 2020; Sorsa et al., 2016). Nevertheless, the main interest in this study was to focus on the correlation between different MMP‐8 forms and species in WB and quantitative aMMP‐8 measurement (IFMA).

We conclude that fragmented MMP‐8 species assessed by monoclonal antibodies are correlating with quantitative aMMP‐8 measurements, as also found previously with an independent polyclonal antibody (Gürsoy et al., 2018), and there is a negative correlation between fragment and complex formation. Different environmental and patient‐related factors (smoking, age, proteolytic/PMN elastase activity) may modify the protein turnover in oral fluids and may cause some variability in quantitative aMMP‐8 measurements. An interesting new finding was that age is possibly related to fragment and complex formation, which may also further modify quantitative MMP‐8 measurement. Whether age is just a periodontitis‐related confounding factor or has some direct immunosenescence‐related mechanism affecting MMP‐8 fragmentation/complex formation, it cannot be answered with the explorative design of this study and needs to be validated with further clinical studies focusing more specifically on age‐related changes in oral fluid biomarker levels.

## AUTHOR CONTRIBUTIONS

Minna‐Maija Ahonen and Veli‐Jukka Uitto designed the original study design and organized the clinical examinations and oral rinse sample collections. Timo Sorsa, Päivi Mäntylä, and Taina Tervahartiala designed and performed all aMMP‐8‐related laboratory analyses. Jussi M. Leppilahti, Päivi Mäntylä, Hannu Kautiainen, and Ismo Räisänen made the statistical analysis and interpretation of data. All authors made substantial contributions to drafting and revising the manuscript and have approved the final version of it.

## CONFLICT OF INTEREST STATEMENT

Timo Sorsa is an inventor of US‐patents 20170023571A1 (granted 6.6.2019), WO 2018/060553 A1 (granted 31.5.2018), 10 488 415 B2, a Japanese patent 2016‐554676 and patent application No. 10‐2016‐7025378 in South Korea (report grant notification, due 25.6.2021). Päivi Mäntylä is an inventor of US‐patent 20170023571A1.

## Data Availability

Research data are not shared.

## References

[cre2803-bib-0001] Arias‐Bujanda, N. , Regueira‐Iglesias, A. , Balsa‐Castro, C. , Nibali, L. , Donos, N. , & Tomás, I. (2019). Accuracy of single molecular biomarkers in gingival crevicular fluid for the diagnosis of periodontitis: A systematic review and meta‐analysis. Journal of Clinical Periodontology, 46(12), 1166–1182. 10.1111/jcpe.13188 31444912

[cre2803-bib-0002] Arias‐Bujanda, N. , Regueira‐Iglesias, A. , Balsa‐Castro, C. , Nibali, L. , Donos, N. , & Tomás, I. (2020). Accuracy of single molecular biomarkers in saliva for the diagnosis of periodontitis: A systematic review and meta‐analysis. Journal of Clinical Periodontology, 47(1), 2–18. 10.1111/jcpe.13202 31560804

[cre2803-bib-0003] Buduneli, E. , Mäntylä, P. , Emingil, G. , Tervahartiala, T. , Pussinen, P. , Barış, N. , Akıllı, A. , Atilla, G. , & Sorsa, T. (2011). Acute myocardial infarction is reflected in salivary matrix metalloproteinase‐8 activation level. Journal of Periodontology, 82(5), 716–725. 10.1902/jop.2010.100492 21091346

[cre2803-bib-0004] Chen, H. Y. , Cox, S. W. , Eley, B. M. , Mäntylä, P. , Rönkä, H. , & Sorsa, T. (2000). Matrix metalloproteinase‐8 levels and elastase activities in Gingival Crevicular Fluid from chronic adult periodontitis patients. Journal of Clinical Periodontology, 27(5), 366–369. 10.1034/j.1600-051x.2000.027005366.x 10847542

[cre2803-bib-0005] DeCarlo, A. A. , Windsor, L. J. , Bodden, M. K. , Harber, G. J. , Birkedal‐Hansen, B. , & Birkedal‐Hansen, H. (1997). Activation and novel processing of matrix metalloproteinases by a thiol‐proteinase from the oral anaerobe *Porphyromonas gingivalis* . Journal of Dental Research, 76(6), 1260–1270. 10.1177/00220345970760060501 9168859

[cre2803-bib-0006] Delima, A. J. , & van Dyke, T. E. (2003). Origin and function of the cellular components in gingival crevice fluid. Periodontology 2000, 31, 55–76. 10.1034/j.1600-0757.2003.03105.x 12656996

[cre2803-bib-0007] Deng, K. , Pelekos, G. , Jin, L. , & Tonetti, M. S. (2021). Diagnostic accuracy of a point‐of‐care aMMP‐8 test in the discrimination of periodontal health and disease. Journal of Clinical Periodontology, 48(8), 1051–1065. 10.1111/jcpe.13485 33998040 PMC8362205

[cre2803-bib-0008] Deng, K. , Wei, S. , Xu, M. , Shi, J. , Lai, H. , & Tonetti, M. S. (2022). Diagnostic accuracy of active matrix metalloproteinase‐8 point‐of‐care test for the discrimination of periodontal health status: Comparison of saliva and oral rinse samples. Journal of Periodontal Research, 57(4), 768–779. 10.1111/jre.12999 35575900

[cre2803-bib-0009] Ding, Y. , Haapasalo, M. , Kerosuo, E. , Lounatmaa, K. , Kotiranta, A. , & Sorsa, T. (1997). Release and activation of human neutrophil matrix metallo‐ and serine proteinases during phagocytosis of *Fusobacterium nucleatum*, *Porphyromonas gingivalis* and *Treponema denticola* . Journal of Clinical Periodontology, 24(4), 237–248. 10.1111/j.1600-051x.1997.tb01837.x 9144046

[cre2803-bib-0010] Ding, Y. , Uitto, V. J. , Firth, J. , Salo, T. , Haapasalo, M. , Konttinen, Y. , & Sorsa, T. (1995). Modulation of host matrix metalloproteinases by bacterial virulence factors relevant in human periodontal diseases. Oral Diseases, 1(4), 279–286. 10.1111/j.1601-0825.1995.tb00194.x 8705837

[cre2803-bib-0011] Ding, Y. , Uitto, V. J. , Haapasalo, M. , Lounatmaa, K. , Konttinen, Y. T. , Salo, T. , Grenier, D. , & Sorsa, T. (1996). Membrane components of *Treponema denticola* trigger proteinase release from human polymorphonuclear leukocytes. Journal of Dental Research, 75(12), 1986–1993. 10.1177/00220345960750121101 9033454

[cre2803-bib-0012] Grigoriadis, A. , Sorsa, T. , Räisänen, I. , Pärnänen, P. , Tervahartiala, T. , & Sakellari, D. (2019). Prediabetes/diabetes can be screened at the dental office by a low‐cost and Fast Chair‐Side/Point‐of‐Care aMMP‐8 immunotest. Diagnostics, 9(4), 151. 10.3390/diagnostics9040151 31627410 PMC6963402

[cre2803-bib-0013] Gursoy, U. K. , Könönen, E. , Pradhan‐Palikhe, P. , Tervahartiala, T. , Pussinen, P. J. , Suominen‐Taipale, L. , & Sorsa, T. (2010). Salivary MMP‐8, TIMP‐1, and ICTP as markers of advanced periodontitis: MMP‐8 in periodontitis. Journal of Clinical Periodontology, 37(6), 487–493. 10.1111/j.1600-051X.2010.01563.x 20507371

[cre2803-bib-0014] Gürsoy, U. K. , Könönen, E. , Tervahartiala, T. , Gürsoy, M. , Pitkänen, J. , Torvi, P. , Suominen, A. L. , Pussinen, P. , & Sorsa, T. (2018). Molecular forms and fragments of salivary MMP‐8 in relation to periodontitis. Journal of Clinical Periodontology, 45(12), 1421–1428. 10.1111/jcpe.13024 30341955

[cre2803-bib-0015] Hanemaaijer, R. , Sorsa, T. , Konttinen, Y. T. , Ding, Y. , Sutinen, M. , Visser, H. , van Hinsbergh, V. W. M. , Helaakoski, T. , Kainulainen, T. , Rönkä, H. , Tschesche, H. , & Salo, T. (1997). Matrix Metalloproteinase‐8 is expressed in rheumatoid synovial fibroblasts and endothelial cells. Journal of Biological Chemistry, 272(50), 31504–31509. 10.1074/jbc.272.50.31504 9395486

[cre2803-bib-0016] Hazeldine, J. , Harris, P. , Chapple, I. L. , Grant, M. , Greenwood, H. , Livesey, A. , Sapey, E. , & Lord, J. M. (2014). Impaired neutrophil extracellular trap formation: A novel defect in the innate immune system of aged individuals. Aging cell, 13(4), 690–698. 10.1111/acel.12222 24779584 PMC4326942

[cre2803-bib-0017] Heikkinen, A. M. , Nwhator, S. O. , Rathnayake, N. , Mäntylä, P. , Vatanen, P. , & Sorsa, T. (2016). Pilot study on oral health status as assessed by an active matrix metalloproteinase‐8 chairside mouthrinse test in adolescents. Journal of Periodontology, 87(1), 36–40. 10.1902/jop.2015.150377 26430926

[cre2803-bib-0018] Hemmilä, I. , Dakubu, S. , Mukkala, V. M. , Siitari, H. , & Lövgren, T. (1984). Europium as a label in time‐resolved immunofluorometric assays. Analytical Biochemistry, 137(2), 335–343. 10.1016/0003-2697(84)90095-2 6375455

[cre2803-bib-0019] Hernández, M. , Gamonal, J. , Tervahartiala, T. , Mäntylä, P. , Rivera, O. , Dezerega, A. , Dutzan, N. , & Sorsa, T. (2010). Associations between matrix metalloproteinase‐8 and ‐14 and myeloperoxidase in gingival crevicular fluid from subjects with progressive chronic periodontitis: A longitudinal study. Journal of Periodontology, 81(11), 1644–1652. 10.1902/jop.2010.100196 20653434

[cre2803-bib-0020] Hirschfeld, J. , Dommisch, H. , Skora, P. , Horvath, G. , Latz, E. , Hoerauf, A. , Waller, T. , Kawai, T. , Jepsen, S. , Deschner, J. , & Bekeredjian‐Ding, I. (2015). Neutrophil extracellular trap formation in supragingival biofilms. International Journal of Medical Microbiology, 305(4–5), 453–463. 10.1016/j.ijmm.2015.04.002 25959370

[cre2803-bib-0021] Kiili, M. , Cox, S. W. , Chen, H. W. , Wahlgren, J. , Maisi, P. , Eley, B. M. , Salo, T. , & Sorsa, T. (2002). Collagenase‐2 (MMP‐8) and collagenase‐3 (MMP‐13) in adult periodontitis: Molecular forms and levels in gingival crevicular fluid and immunolocalisation in gingival tissue. Journal of Clinical Periodontology, 29(3), 224–232. 10.1034/j.1600-051x.2002.290308.x 11940142

[cre2803-bib-0022] Knauper, V. , Kramer, S. , Reinke, H. , & Tscheche, H. (1990). Characterization and activation of procollagenase from human polymorphonuclear leucocytes. N‐terminal sequence determination of the proenzyme and various proteolytically activated forms. European Journal of Biochemistry, 189(2), 295–300. 10.1111/j.1432-1033.1990.tb15489.x 2159879

[cre2803-bib-0023] Knäuper, V. , Osthues, A. , DeClerck, Y. A. , Langley, K. E. , Bläser, J. , & Tschesche, H. (1993). Fragmentation of human polymorphonuclear‐leucocyte collagenase. Biochemical Journal, 291(Pt 3), 847–854. 10.1042/bj2910847 8489511 PMC1132446

[cre2803-bib-0024] Lahdentausta, L. , Paju, S. , Mäntylä, P. , Buhlin, K. , Pietiäinen, M. , Tervahartiala, T. , Nieminen, M. S. , Sinisalo, J. , Sorsa, T. , & Pussinen, P. J. (2019). Smoking confounds the periodontal diagnostics using saliva biomarkers. Journal of Periodontology, 90(5), 475–483. 10.1002/JPER.18-0545 30447005

[cre2803-bib-0025] Lähteenmäki, H. , Tervahartiala, T. , Räisänen, I. T. , Pärnänen, P. , Mauramo, M. , Gupta, S. , Sampson, V. , Rathnayake, N. , Heikkinen, A. M. , Alassiri, S. , Gieselmann, D. R. , Frankenberger, R. , & Sorsa, T. (2022). Active MMP‐8 point‐of‐care (PoC)/chairside enzyme‐test as an adjunctive tool for early and real‐time diagnosis of peri‐implantitis. Clinical and Experimental Dental Research, 8(2), 485–496. 10.1002/cre2.537 35118828 PMC9033547

[cre2803-bib-0026] Lauhio, A. , Salo, T. , Ding, Y. , Konttinen, Y. T. , Nordström, D. , Tschesche, H. , Lähdevirta, J. , Golub, L. M. , & Sorsa, T. (1994). In vivo inhibition of human neutrophil collagenase (MMP‐8) activity during long‐term combination therapy of doxycycline and non‐steroidal anti‐inflammatory drugs (NSAID) in acute reactive arthritis. Clinical & Experimental Immunology, 98(1), 21–28. 10.1111/j.1365-2249.1994.tb06601.x 7923879 PMC1534162

[cre2803-bib-0027] Lauhio, A. , Sorsa, T. , Srinivas, R. , Stenman, M. , Tervahartiala, T. , Stenman, U. H. , Grönhagen‐Riska, C. , & Honkanen, E. (2008). Urinary matrix metalloproteinase −8, −9, −14 and their regulators (TRY‐1, TRY‐2, TATI) in patients with diabetic nephropathy. Annals of Medicine, 40(4), 312–320. 10.1080/07853890801923746 18428024

[cre2803-bib-0028] Leppilahti, J. , Ahonen, M. M. , Hernández, M. , Munjal, S. , Netuschil, L. , Uitto, V. J. , Sorsa, T. , & Mäntylä, P. (2011). Oral rinse MMP‐8 point‐of‐care immuno test identifies patients with strong periodontal inflammatory burden. Oral Diseases, 17(1), 115–122. 10.1111/j.1601-0825.2010.01716.x 20659259

[cre2803-bib-0029] Leppilahti, J. M. , Hernández‐Ríos, P. A. , Gamonal, J. A. , Tervahartiala, T. , Brignardello‐Petersen, R. , Mantyla, P. , Sorsa, T. , & Hernández, M. (2014). Matrix metalloproteinases and myeloperoxidase in gingival crevicular fluid provide site‐specific diagnostic value for chronic periodontitis. Journal of Clinical Periodontology, 41(4), 348–356. 10.1111/jcpe.12223 24382144

[cre2803-bib-0030] Lorenz, K. , Keller, T. , Noack, B. , Freitag, A. , Netuschil, L. , & Hoffmann, T. (2017). Evaluation of a novel point‐of‐care test for active matrix metalloproteinase‐8: Agreement between qualitative and quantitative measurements and relation to periodontal inflammation. Journal of Periodontal Research, 52(2), 277–284. 10.1111/jre.12392 27214099

[cre2803-bib-0031] Määttä, M. , Kari, O. , Tervahartiala, T. , Peltonen, S. , Kari, M. , Saari, M. , & Sorsa, T. (2006). Tear fluid levels of MMP‐8 are elevated in ocular rosacea—Treatment effect of oral doxycycline. Graefe's Archive for Clinical and Experimental Ophthalmology, 244(8), 957–962. 10.1007/s00417-005-0212-3 16411105

[cre2803-bib-0032] Mäntylä, P. , Stenman, M. , Kinane, D. , Salo, T. , Suomalainen, K. , Tikanoja, S. , & Sorsa, T. (2006). Monitoring periodontal disease status in smokers and nonsmokers using a gingival crevicular fluid matrix metalloproteinase‐8‐specific chair‐side test. Journal of Periodontal Research, 41(6), 503–512. 10.1111/j.1600-0765.2006.00897.x 17076774

[cre2803-bib-0033] Mäntylä, P. , Stenman, M. , Kinane, D. F. , Tikanoja, S. , Luoto, H. , Salo, T. , & Sorsa, T. (2003). Gingival crevicular fluid collagenase‐2 (MMP‐8) test stick for chair‐side monitoring of periodontitis. Journal of Periodontal Research, 38(4), 436–439. 10.1034/j.1600-0765.2003.00677.x 12828663

[cre2803-bib-0034] Nieminen, A. , Nordlund, L. , & Uitto, V. J. (1993). The effect of treatment on the activity of salivary proteases and glycosidases in adults with advanced periodontitis. Journal of Periodontology, 64(4), 297–301. 10.1902/jop.1993.64.4.297 8483092

[cre2803-bib-0035] Nieminen, M. T. , Listyarifah, D. , Hagström, J. , Haglund, C. , Grenier, D. , Nordström, D. , Uitto, V. J. , Hernandez, M. , Yucel‐Lindberg, T. , Tervahartiala, T. , Ainola, M. , & Sorsa, T. (2018). Treponema denticola chymotrypsin‐like proteinase may contribute to orodigestive carcinogenesis through immunomodulation. British Journal of Cancer, 118(3), 428–434. 10.1038/bjc.2017.409 29149107 PMC5808028

[cre2803-bib-0036] Nwomeh, B. C. , Liang, H. X. , Cohen, I. K. , & Yager, D. R. (1999). MMP‐8 is the predominant collagenase in healing wounds and nonhealing ulcers. Journal of Surgical Research, 81(2), 189–195. 10.1006/jsre.1998.5495 9927539

[cre2803-bib-0037] Owen, C. A. , Hu, Z. , Lopez‐Otin, C. , & Shapiro, S. D. (2004). Membrane‐Bound matrix metalloproteinase‐8 on activated polymorphonuclear cells is a potent, tissue inhibitor of metalloproteinase‐resistant collagenase and serpinase. The Journal of Immunology, 172(12), 7791–7803. 10.4049/jimmunol.172.12.7791 15187163

[cre2803-bib-0038] Pirilä, E. , Korpi, J. T. , Korkiamäki, T. , Jahkola, T. , Gutierrez‐Fernandez, A. , Lopez‐Otin, C. , Saarialho‐Kere, U. , Salo, T. , & Sorsa, T. (2007). Collagenase‐2 (MMP‐8) and matrilysin‐2 (MMP‐26) expression in human wounds of different etiologies. Wound Repair and Regeneration, 15(1), 47–57. 10.1111/j.1524-475X.2006.00184.x 17244319

[cre2803-bib-0039] Preshaw, P. M. , Henne, K. , Taylor, J. J. , Valentine, R. A. , & Conrads, G. (2017). Age‐related changes in immune function (immune senescence) in caries and periodontal diseases: A systematic review. Journal of Clinical Periodontology, 44, 44. 10.1111/jcpe.12675 28266110

[cre2803-bib-0040] Räisänen, I. , Sorsa, T. , van der Schoor, G. J. , Tervahartiala, T. , van der Schoor, P. , Gieselmann, D. R. , & Heikkinen, A. (2019). Active matrix metalloproteinase‐8 point‐of‐care (PoC)/chairside mouthrinse test vs. bleeding on probing in diagnosing subclinical periodontitis in adolescents. Diagnostics, 9(1), 34. 10.3390/diagnostics9010034 30909530 PMC6468891

[cre2803-bib-0041] Räisänen, I. T. , Lähteenmäki, H. , Gupta, S. , Grigoriadis, A. , Sahni, V. , Suojanen, J. , Seppänen, H. , Tervahartiala, T. , Sakellari, D. , & Sorsa, T. (2021). An aMMP‐8 point‐of‐care and questionnaire based real‐time diagnostic toolkit for medical practitioners. Diagnostics, 11(4), 711. 10.3390/DIAGNOSTICS11040711 33921148 PMC8071538

[cre2803-bib-0042] Rautava, J. , Gürsoy, U. K. , Kullström, A. , Könönen, E. , Sorsa, T. , Tervahartiala, T. , & Gürsoy, M. (2020). An oral rinse active matrix metalloproteinase‐8 point‐of‐care immunotest may be less accurate in patients with Crohn's disease. Biomolecules, 10(3), 395. 10.3390/biom10030395 32143418 PMC7175303

[cre2803-bib-0043] Romanelli, R. , Mancini, S. , Laschinger, C. , Overall, C. M. , Sodek, J. , & McCulloch, C. A. G. (1999). Activation of neutrophil collagenase in periodontitis. Infection and Immunity, 67(5), 2319–2326. 10.1128/iai.67.5.2319-2326.1999 10225890 PMC115973

[cre2803-bib-0044] Saari, H. , Suomalainen, K. , Lindy, O. , Konttinen, Y. , & Sorsa, T. (1990). Activation of latent human neutrophil collagenase by reactive oxygen species and serine proteases. Biochemical and Biophysical Research Communications, 171(3), 979–987. 10.1016/0006-291x(90)90780-q 2171513

[cre2803-bib-0045] Soloman, S. R. , & Sawilowsky, S. S. (2009). Impact of rank‐based normalizing transformations on the accuracy of test scores. Journal of Modern Applied Statistical Methods, 8(2), 448–462. 10.22237/jmasm/1257034080

[cre2803-bib-0046] Sorsa, T. , Alassiri, S. , Grigoriadis, A. , Räisänen, I. T. , Pärnänen, P. , Nwhator, S. O. , Gieselmann, D. R. , & Sakellari, D. (2020). Active MMP‐8 (AMMP‐8) as a grading and staging biomarker in the periodontitis classification. Diagnostics, 10(2), 61. 10.3390/diagnostics10020061 31979091 PMC7168924

[cre2803-bib-0047] Sorsa, T. , Ding, Y. L. , Ingman, T. , Salo, T. , Westerlund, U. , Haapasalo, M. , Tschesche, H. , & Konttinen, Y. T. (1995). Cellular source, activation and inhibition of dental plaque collagenase. Journal of Clinical Periodontology, 22(9), 709–717. 10.1111/j.1600-051x.1995.tb00831.x 7593702

[cre2803-bib-0048] Sorsa, T. , Gursoy, U. K. , Nwhator, S. , Hernandez, M. , Tervahartiala, T. , Leppilahti, J. , Gursoy, M. , Könönen, E. , Emingil, G. , Pussinen, P. J. , & Mäntylä, P. (2016). Analysis of matrix metalloproteinases, especially MMP‐8, in gingival crevicular fluid, mouthrinse and saliva for monitoring periodontal diseases. Periodontology 2000, 70(1), 142–163. 10.1111/prd.12101 26662488

[cre2803-bib-0049] Sorsa, T. , HernÃ¡ndez, M. , Leppilahti, J. , Munjal, S. , Netuschil, L. , & MÃ¤ntylÃ¤, P. (2010). Detection of gingival crevicular fluid MMP‐8 levels with different laboratory and chair‐side methods. Oral Diseases, 16(1), 39–45. 10.1111/j.1601-0825.2009.01603.x 19627514

[cre2803-bib-0050] Sorsa, T. , Ingman, T. , Suomalainen, K. , Haapasalo, M. , Konttinen, Y. T. , Lindy, O. , Saari, H. , & Uitto, V. J. (1992). Identification of proteases from periodontopathogenic bacteria as activators of latent human neutrophil and fibroblast‐type interstitial collagenases. Infection and Immunity, 60(11), 4491–4495. 10.1128/iai.60.11.4491-4495.1992 1398963 PMC258193

[cre2803-bib-0051] Sorsa, T. , Mäntylä, P. , Rönkä, H. , Kallio, P. , Kallis, G. B. , Lundqvist, C. , Kinane, D. F. , Salo, T. , Golub, L. M. , Teronen, O. , & Tikanoja, S. (1999). Scientific basis of a matrix metalloproteinase‐8 specific chair‐side test for monitoring periodontal and peri‐implant health and disease. Annals of the New York Academy of Sciences, 878, 130–140. 10.1111/j.1749-6632.1999.tb07679.x 10415725

[cre2803-bib-0052] Sorsa, T. , Nwhator, S. O. , Sakellari, D. , Grigoriadis, A. , Umeizudike, K. A. , Brandt, E. , Keskin, M. , Tervahartiala, T. , Pärnänen, P. , Gupta, S. , Mohindra, R. , Bostanci, N. , Buduneli, N. , & Räisänen, I. T. (2022). aMMP‐8 oral fluid PoC test in relation to oral and systemic diseases. Frontiers in oral health, 3), 897115. 10.3389/froh.2022.897115 35757444 PMC9226345

[cre2803-bib-0053] Sorsa, T. , Sahni, V. , Buduneli, N. , Gupta, S. , Räisänen, I. T. , Golub, L. M. , Lee, H. M. , Pätilä, T. , Bostanci, N. , Meurman, J. , Pärnänen, P. , Nwhator, S. O. , Singla, M. , & Gauba, K. (2021). Active matrix metalloproteinase‐8 (aMMP‐8) point‐of‐care test (POCT) in the COVID‐19 pandemic. Expert Review of Proteomics, 18(8), 707–717. 10.1080/14789450.2021.1976151 34468272 PMC8442753

[cre2803-bib-0054] Sorsa, T. , Suomalainen, K. , Turto, H. , & Lindy, S. (1985). Partial purification and characterization of latent human leukocyte collagenase. Medical Biology, 63(2), 66–72. 2999523

[cre2803-bib-0055] Sorsa, T. , Uitto, V. J. , Suomalainen, K. , Vauhkonen, M. , & Lindy, S. (1988). Comparison of interstitial collagenases from human gingiva, sulcular fluid and polymorphonuclear leukocytes. Journal of Periodontal Research, 23(6), 386–393. 10.1111/j.1600-0765.1988.tb01618.x 2851042

[cre2803-bib-0056] Uitto, V. J. , Nieminen, A. , Coil, J. , Hurttia, H. , & Larjava, H. (1996). Oral fluid elastase as an indicator of periodontal health. Journal of Clinical Periodontology, 23(1), 30–37. 10.1111/j.1600-051x.1996.tb00501.x 8636454

[cre2803-bib-0057] Uitto, V. J. , Suomalainen, K. , & Sorsa, T. (1990). Salivary collagenase. Origin, characteristics and relationship to periodontal health. Journal of Periodontal Research, 25(3), 135–142. 10.1111/j.1600-0765.1990.tb01035.x 2163444

[cre2803-bib-0058] Uitto, V. J. , Turto, H. , & Saxen, L. (1978). Extraction of collagenase from human gingiva. Journal of Periodontal Research, 13(3), 207–214. 10.1111/j.1600-0765.1978.tb00172.x 207848

[cre2803-bib-0059] Wang, X. , Rojas‐Quintero, J. , Wilder, J. , Tesfaigzi, Y. , Zhang, D. , & Owen, C. A. (2019). Tissue inhibitor of metalloproteinase‐1 promotes polymorphonuclear neutrophil (PMN) pericellular proteolysis by anchoring matrix metalloproteinase‐8 and ‐9 to PMN surfaces. The Journal of Immunology, 202(11), 3267–3281. 10.4049/jimmunol.1801466 31019060 PMC7347292

[cre2803-bib-0060] van Wart, H. E. (1992). Human neutrophil collagenase. Matrix Supplement, 1, 31–36. 1480044

